# Uncovering multilevel drivers of cancer disparities among Latinos in the United States

**DOI:** 10.3389/fpubh.2025.1591074

**Published:** 2025-07-23

**Authors:** Amelie G. Ramirez, Edgar Munoz, Lorna Rodriguez-Rodriguez, Leon Bernal-Mizrachi, Patricia Chalela, Jose Aron Lopez, Paulo S. Pinheiro, Barbara Segarra-Vasquez, Gregory Talavera, Luis G. Carvajal-Carmona, Adolfo Diaz Duque, Cliff Despres, Edward J. Trapido

**Affiliations:** ^1^The University of Texas Health Science Center at San Antonio, San Antonio, TX, United States; ^2^City of Hope National Medical Center, Duarte, CA, United States; ^3^Emory University, Atlanta, GA, United States; ^4^University of Washington, Seattle, WA, United States; ^5^University of Miami, Coral Gables, FL, United States; ^6^University of Puerto Rico, San Juan, Puerto Rico; ^7^San Diego State University, San Diego, CA, United States; ^8^University of California, Davis, Davis, CA, United States; ^9^Louisiana State University School of Public Health, New Orleans, LA, United States

**Keywords:** cancer disparities, Latinos, health equity, cultural barriers, clinical trial diversity, environmental health

## Abstract

Cancer disparities among Latinos in the United States persist as a significant public health challenge, characterized by inequitable outcomes throughout the cancer continuum. Latinos experience elevated rates and poorer prognoses in certain cancers compared to other populations, driven by complex, multilevel interactions involving environmental exposures, genetic factors, cultural practices, and systemic inequalities. Recognizing the substantial heterogeneity among Latinos—including differences in national origin, immigration experiences, socioeconomic status, language, and race—is crucial, as oversimplification risks masking key disparities. To comprehensively understand these dynamics, the Task Force: Latino Researchers Against Cancer (TFLRAC) convened specialists in oncology, public health, and behavioral sciences to systematically examine the multiple influences contributing to these disparities. Findings highlight environmental hazards, genetic susceptibilities, cultural stigmas, and linguistic barriers as significant drivers. Systemic issues such as discrimination, colorism, inadequate healthcare coverage, workforce disparities, socioeconomic challenges, and underrepresentation in clinical trials further compound these inequities. Addressing these disparities requires a multifaceted strategy, including targeted research, culturally tailored interventions, and comprehensive policy reforms to improve healthcare access, workforce diversity, and clinical trial inclusion. Collaborative efforts across academia, healthcare systems, community organizations, governmental agencies, and industry partners are imperative to achieve equitable cancer outcomes among Latinos.

## Introduction

The Latino population is the largest minority in the United States (US), with 63.7 million individuals accounting for 19.1% of the total US population (1). It is important to recognize that “Latino” is an ethno-racial category primarily used for demographic and public health purposes within the US context. This socio-political construct encompasses a highly diverse population with varied ancestries, cultural heritages, countries of origin (such as Mexican, Cuban, Puerto Rican, Salvadoran, Dominican, Colombian, and others), immigration histories, languages, generational statuses, and socioeconomic conditions—differences that significantly influence health outcomes.

Spanish is widely spoken in Latino homes and is the second-most common language in the US (2). The Latino community experiences lower educational attainment and reduced median income, with a higher rate of poverty compared to the general population. Cancer disproportionately affects the Latino population and is the leading cause of death in the US Latino community, making cancer a critical issue to address in this growing population. An estimated 176,600 new cancer cases and 46,500 cancer deaths were expected to occur among US Latino individuals in 2021. Breast cancer is the leading cause of cancer death among US Latina women, and lung cancer is the leading cause of cancer death among Latino men (3).

Compared to non-Hispanic White patients, Latino men and women have higher rates of some less common cancers, such as gallbladder cancer and infection-related cancers of the liver, stomach, and cervix (3). Latino children also have the highest rates of acute lymphoblastic leukemia, the most common cancer in children (4). Cancer is often diagnosed at a later stage in Latino patients, making treatment more challenging, and they are less likely to receive timely and appropriate care (3). Participation in clinical trials is also lower among Latino patients, contributing to reduced survival rates for some cancers. However, cancer incidence and mortality vary widely within the heterogeneity Latino population (5), highlighting the need to identify specific drivers of these disparities and propose targeted solutions.

This heterogeneity extends across national origins, socioeconomic statuses, immigration backgrounds, and notably, racial and phenotypic diversity, including individuals who identify as Afro-Latino (6–9). Acknowledging this diversity—particularly differences in racial identity and skin tone—is essential, as these factors shape unique experiences of discrimination and health inequity, especially among subgroups affected by colorism. Understanding these internal differences and disaggregating data accordingly is crucial for identifying specific health burdens (10–12).

Although this article does not present new empirical data, it synthesizes consensus perspectives from Latino experts with lived experience, cultural fluency, and domain expertise in Latino cancer disparities. The qualitative insights reflect community-grounded understandings informed by extensive research, clinical practice, and policy engagement. As a Perspective Article, it aims to frame current issues, identify research gaps, and call for future empirical and qualitative research—including patient testimonies, ethnographic inquiry, and community-based research—to further develop and validate the framework presented.

## Methods

### Task force: Latino researchers against cancer

Considering the current state of the Latino cancer experience, a task force was established (Task Force: Latino Researchers Against Cancer, TFLRAC) with the goal to explore the causes and multilevel drivers of health impacting the Latino cancer experience.

Throughout 2023, the Task Force conducted structured meetings, focusing on distinct elements of the Latino cancer continuum. These sessions featured detailed agendas and facilitated in-depth discussions among members, who brought specialized expertise and diverse informational sources. This format ensured a focused examination of cancer disparities within the Latino community, allowing for an effective integration of insights and resources pertinent to the task force’s objectives.

Rather than collecting new empirical data, this process prioritized knowledge integration from existing literature, clinical experience, and community engagement. Members of TFLRAC include Latino scholars, clinicians, and public health professionals with cultural familiarity and insider perspectives. The collaborative nature of these discussions enabled the distillation of key themes and contextual factors affecting Latino cancer care, which form the basis of this Perspective.

## Results

**Figure 1:**
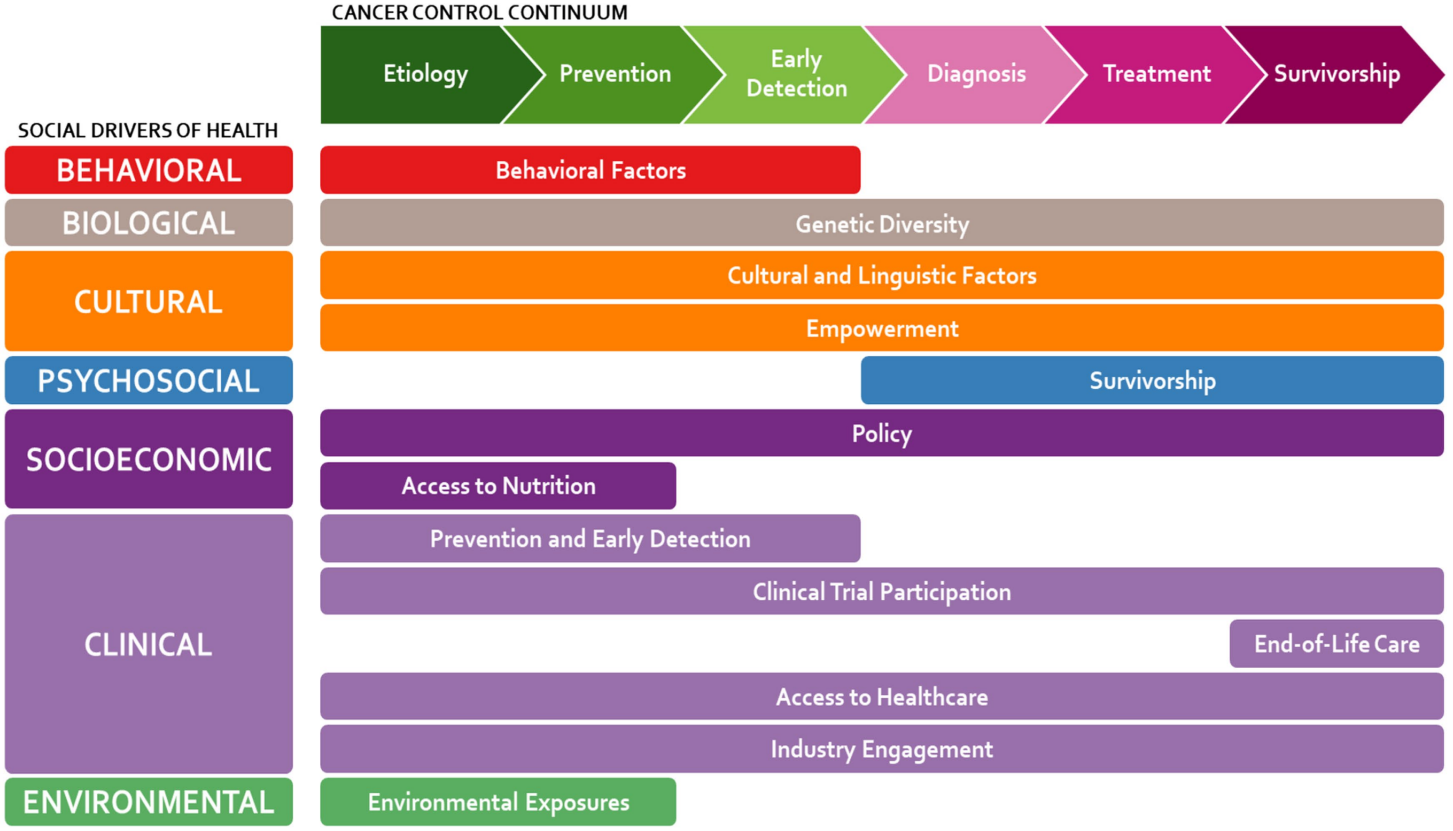
Factors affecting the state of cancer among Latinos, identified by the task force: Latino researchers against cancer (TFLRAC).

The TFLRAC identified environmental, genetic, cultural, and linguistic, behavioral, and systemic and access-related factors contributing to the Latino cancer experience. Figure 1 illustrates these factors mapped across the cancer control continuum and categorized by overarching social drivers of health, providing a visual framework for the subsequent discussion.

As each topic was discussed, two main questions pertaining to Latino cancer inequity and solutions-based interventions were considered:

1. Why do we think cancer has become the leading cause of mortality among US Latinos?
2. How can we address the unique cultural, linguistic, and socioeconomic barriers contributing to disparities in cancer incidence, mortality, and survivorship among US Latinos?

The discussion for each topic is summarized.

### Environmental exposures

Environmental factors are a major cause of cancer disparities in Latino communities. Many Latino communities are in areas with higher pollution rates leading to elevated cancer risk. Latinos are exposed to higher levels of dangerous fine particulate air pollution and live in some of the most ozone-polluted cities (13, 14). The interplay between rising temperatures and altered precipitation patterns due to climate change profoundly impacts water quality. Living near toxic waste sites can also expose individuals to various carcinogens (15).

Many Latinos work in the agriculture (16) and construction industries (17), both of which increase the risk of exposure to carcinogens. Studies have shown that Latino agricultural workers are frequently exposed to insecticides and pesticides (18) Aflatoxin, another environmental toxin found in Latin America and southern US. can increase cancer risk (19) Other environmental factors include second-hand smoke and limited access to green spaces (20).

Climate change is a major contributor to increased environmental exposure among Latino individuals, who often live in areas where severe effects of climate change occur: droughts and fires, record-breaking heat, and hurricanes (21).

Despite what is known about the connection between environmental factors and cancer among Latino populations, there is still a missing link in many specific situations, highlighting the need for more research. The association between pesticide exposure and cancer in largely Latino workforces may differ across varied working conditions, climates, local regulations, and worker heritage. Other associations are clearer, such as increased rates of lung cancer associated with poor air quality due to wildfires. Chemicals in some plastics may increase the risk of breast cancer. Much more research is needed to fully understand these connections.

### Genetic diversity

The genetic diversity among Latinos is vast, with various degrees of Native, African, and European ancestry represented in different populations. Genetic origin may be associated with higher risk of certain cancers. Higher rates of nonalcoholic fatty liver disease and alcohol related disease among Latino populations may contribute to a greater risk of liver cancer (22). This increased risk is not homogenous across Latino subgroups and varies based on country of origin (23). The patatin-like phospholipase domain containing 3 (PNPLA3) gene is associated with the development of nonalcoholic fatty liver disease, and the high-risk allele is found more commonly in the Mexican indigenous population (24). Studies have shown genetic heterogeneity with regard to prognosis among Latino gastric cancer patients (25).

### Heterogeneity by country of origin, immigration status, and nativity

Data from population-based cancer registries reveal substantial heterogeneity within the Latino population across multiple dimensions. Subgroups defined by self-reported national origin—such as Mexican, Cuban, Puerto Rican, Central American, and South American—show distinct patterns in cancer incidence, stage at diagnosis, survival, and mortality (26). These differences are further influenced by nativity (US-born vs. foreign-born) (27) and geographic region (California vs. Florida).

### Cultural and linguistic factors

Cultural beliefs and reliance on traditional medicine are major factors in the Latino healthcare experience. Insights presented here are informed by members of our Task Force—Latino cancer experts with both lived experience and cultural familiarity—and reflect emic (insider) perspectives on sociocultural dynamics.

Latino cultural beliefs significantly impact decisions and interactions with the healthcare system (28). Beliefs such as *familismo* (strong family orientation), *fatalismo* (a sense of fatalism regarding illness), *machismo*, and *marianismo* (gender role expectations) have been widely discussed in anthropological and sociological literature as influential factors in Latino health behaviors. These values are not monolithic across the Latino community but vary by geography, generation, migration history, and personal context.

*Familismo* can serve as both a source of support and a source of burden, particularly in resource-limited settings where caregiving responsibilities may delay care-seeking. *Fatalismo*, sometimes viewed as a barrier to preventive care, may coexist with proactive health behaviors when interpreted through a spiritual or faith-based lens. Traditional gender roles associated with *machismo* and *marianismo* can shape attitudes toward medical care and influence when and how care is sought. These patterns require careful navigation by healthcare providers to avoid assumptions.

A lack of education among some foreign-born Latinos and a strong belief in natural medicine may prevent some patients from understanding certain medical treatments, such as chemotherapy, antibody-based therapies, and immunotherapies. Language barriers represent one of the highest hurdles to meaningful cancer care for the Latino population. The entirety of the Latino cancer continuum is impacted by language.

### Behavioral factors

When cultural beliefs collide with American culture, health can be compromised. A decline in physically demanding work, and the increase in availability of processed foods high in fat, sugar, and salt can result in a decrease in overall health of the community. This phenomenon may contribute to certain health issues (29). Unfavorable diets and increasing rates of obesity contribute to the high prevalence of colorectal cancer among Latinos. Increased rates of liver cancer could be associated with increased alcohol consumption (30), drug use and needle sharing (31), and sexually transmitted diseases (32).

Beyond healthy habits, Latino individuals also use less healthcare and social services than the average American. Health insurance is a concern, with difficulties obtaining and retaining insurance. Some Latino individuals prefer to pay cash for health costs and remain suspicious of health insurance companies.

### Access to nutrition

Food sources also play a role in cancer risks. The cascading effect of climate change on agricultural productivity has culminated in diminished access to fresh and nutritious alimentary sources. Diets replete with processed foods and deficient in fruits and vegetables have been linked to an elevated risk of malignancies, notably colorectal and stomach cancers. Latino communities are particularly susceptible to this, And over 10% of Hispanic individuals have difficulty accessing fresh produce (33). This is exacerbated by government subsidies that make unhealthy foods less expensive.

### Access to healthcare

Healthcare itself poses many difficulties for many Latinos, with lack of a diverse, culturally competent workforce, health insurance coverage, and language barriers. There are disproportionately fewer Latino workers across the entire healthcare workforce when compared to the general population, including medical oncologists (34). This lack of representation should be addressed throughout the medical training pipeline. Part of the problem with underrepresentation in healthcare is that tests in the US are given in English. For this reason, there is a great need for Latino practitioners to mentor young students.

Health insurance is also a challenge for many Latinos, with a high percentage lacking health insurance (35). A disruption in coverage for a cancer patient has been shown to adversely affect the receipt of cancer care and survival (36). Furthermore, some states have not expanded Medicaid, resulting in high rates of uninsured individuals (37). The burden of supporting multi-generational families and a distrust of government policy, make the idea of paying for an uncertain future benefit unappealing. Type of insurance also makes a key difference in outcome. Underinsurance can be a big problem, with large copays and coinsurance making costs untenable.

### Clinical trial participation

**Figure 2:**
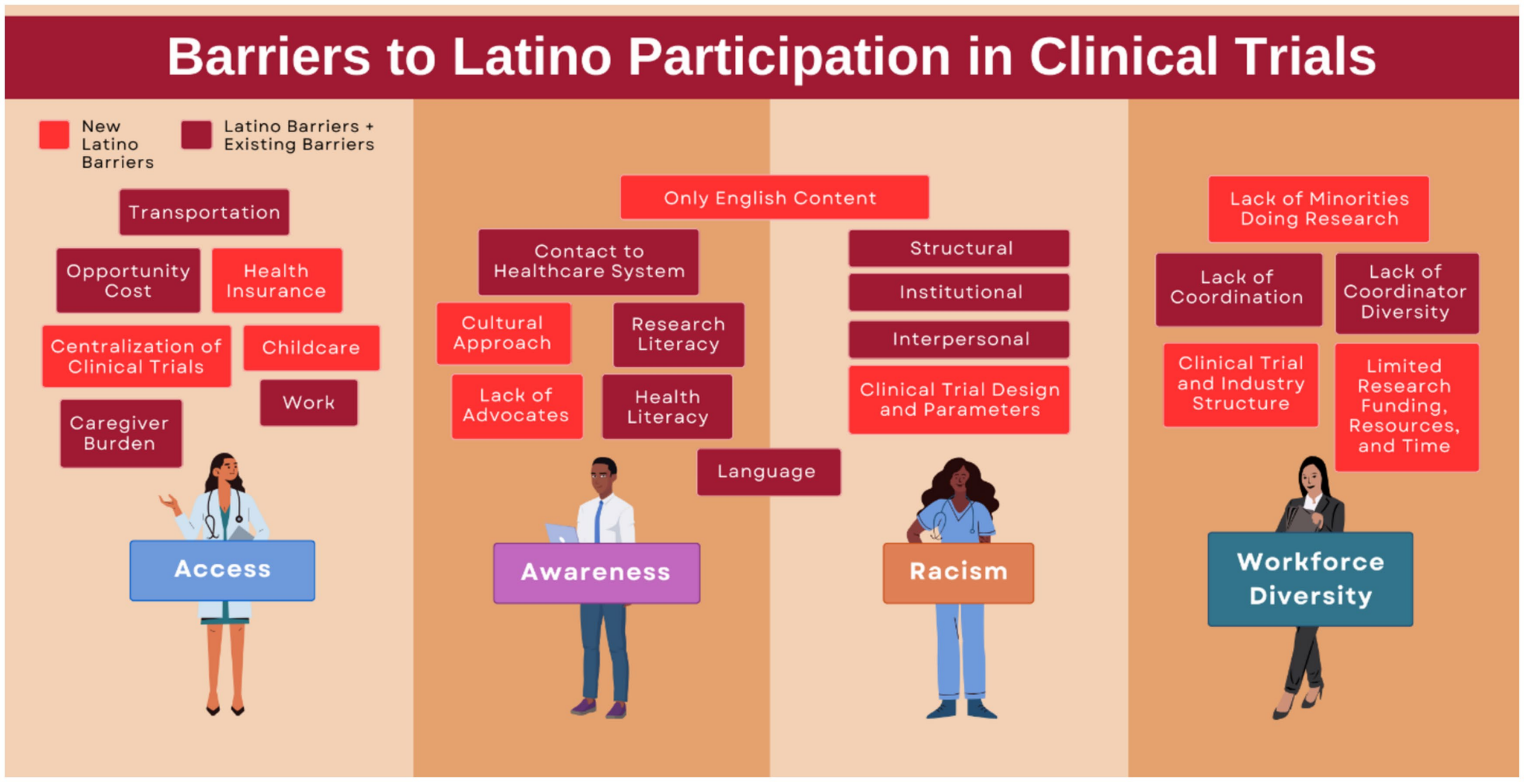
Barriers to Latino participation in clinical trials. Adapted from community engagement and clinical trial diversity: navigating barriers and co-designing solutions – a similar report from the “Health Equity through Diversity” series (38).

There is vast disparity in cancer clinical trial participation among minorities in the US, particularly in the Latino population (38–40). As shown in Figure 2, which is adapted from Reopell et al. (38), these barriers can be broadly categorized into issues of access, awareness, systemic racism, and workforce diversity. Studies show that representation of Hispanic participation was the lowest overall when compared to other racial and ethnic groups (41, 42). Clinical trial design must be methodical and inclusive of diversity among potential minority recruits. Even clinical trials with thousands of Latino participants may yield questionable data if Hispanic/Latino subgroups, ancestry, and culture are not considered.

Clinical trials often require multiple, sometimes lengthy visits, preventing patients from working or caring for family members. This difficulty is exacerbated by limited paid sick days and a lack of medical insurance coverage for some clinical trials. Another barrier may be the lack of Latino patients living in areas surrounding most traditional research centers. Patients also tend to find the idea of clinical trials foreign. Others are ineligible based on stringent exclusion criteria for conditions that are highly prevalent among US-based Latinos (43).

### Prevention and early detection

Patient education, cancer screenings, and enrollment in insurance programs are also best achieved at the community level. Churches and other community organizations, which have already established trust, are currently being used to great effect (44). Some organizations have even begun to focus on health education in elementary schools (45). The main barrier to prevention and early detection strategies is cost.

### Empowerment

Before a discussion can be had concerning Latino empowerment, it is important to note that many Latino patients might not respond positively to the word “empowerment” at all. Instead, “understanding” and “communication” are concepts that might be more readily received. Then, through deeper understanding, patients might become more engaged and take a more active, informed role in their cancer care, making use of available resources to navigate the healthcare system. Cultural fluency training and standardized language training for providers warrant further consideration (46).

### Survivorship

By 2030, the number of cancer survivors in the US is projected to reach 22.2 million, with Latino survivors accounting for approximately 20% of that number (47). This number reflects a rise in cancer survivorship in Latino communities, a testament to advancements in early detection and treatment. However, the post-treatment journey is often complicated by cultural beliefs, language barriers, and socio-economic constraints. Latino cancer survivors are more likely to report suboptimal physical well-being. Health behaviors and access to health care are associated with quality of life (48). The stress of a cancer diagnosis can also result in mental health issues. A cultural stigma may prevent many from seeking help for mental health concerns (49, 50). Like all cancer survivors, Latino patients face potential long-term and late effects of cancer and its treatment. Social support is a crucial component of survivorship, but many Latino patients face the challenge of social isolation. There is a critical need for interventions that support caregivers. However, the lack of bilingual counselors and Spanish-speaking survivor role models in the media can limit openness among survivors. The needs of caregivers are often overlooked, with these individuals carrying the major burdens. These caregivers carry a large psychosocial burden (51). Research is needed to further understand the needs of caregivers and the interventions that could be most impactful (52).

### End-of-life care

Some Latino patients originating from other countries wish to go home at the end of their lives. This presents difficulties for travel and coordination of care in their home countries. A frequently overlooked consequence of this travel is that many US-based Latino cancer patients die in another country, which may affect morbidity and mortality calculations. Palliative care and hospice care are vital for the care of cancer survivors but are often difficult to obtain (53). Insurance coverage for hospice care and home equipment can give patients dignity and relief. Part of the problem for cancer patients trying to obtain hospice care is the difficulty in navigating a disjointed healthcare system. Advanced care planning is often underutilized in Latino patients, partially due to a lack of understanding that these decisions can ease the burden placed on the patient’s family. Latino cultures tend to be less control-focused, and there is often a stigma around discussing end-of-life.

### Policy

Political participation is lower among Latino voters compared to the general population (54). Many of the systemic inequities impacting Latino cancer care are supported by government policy. Some policies are counter to the safety of individuals, such as state-initiated incentives that relax regulations and jeopardize the safety of nearby residents.

## Discussion

The state of cancer in the US Latino population is concerning, and its causes are multifactorial. Our findings align with previous research highlighting a higher incidence of specific cancers in this group, later-stage diagnoses, and a notable absence of targeted research on both factors and outcomes (55, 56). Importantly, Latino communities in the US are internally diverse, encompassing a spectrum of cultural identities, immigration experiences, and socioeconomic conditions. These differences shape cancer risk, presentation, and treatment outcomes.

In its inaugural year, the Task Force: Latino Researchers Against Cancer (TFLRAC) has undertaken a comprehensive exploration of the multifaceted factors influencing cancer disparities among US Latinos. This foundational work has identified key areas such as environmental and genetic influences, cultural and linguistic barriers, health behavior patterns, and systemic and access-related issues.

The task force’s exploration of cultural beliefs, including *familismo*, *fatalismo*, *machismo*, and *marianismo*, emphasized the nuanced ways in which these values inform health decisions. For instance, *familismo* may bolster care adherence through strong social support, yet also discourage individual prioritization of preventive care. *Fatalismo* is not necessarily a passive belief but often integrates spiritual dimensions that can coexist with treatment engagement. A deeper understanding of these dynamics—grounded in insider perspectives and acknowledging their complexity and variation within diverse Latino subgroups—is essential to effective care.

The underrepresentation of Latinos in clinical trials remains a pressing issue, limiting generalizability of findings and contributing to inequities in evidence-based care. Greater attention to inclusion, cultural relevance, and accessibility is necessary in research and care delivery. Immediate efforts must also focus on educating patients about prevention and early detection, improving survivorship resources, and expanding culturally and linguistically aligned support systems.

To effectively address the complexity of cancer disparities within Latino populations, it is critical to disaggregate data by subgroups such as national origin, migration history, primary language, generational status, race, skin color, and socioeconomic background (7, 9, 11, 12). Aggregated data that treats Latinos as a single, uniform group can obscure significant differences in cancer incidence, risk factors, access to care, and treatment outcomes. Without attention to these intra-group variations and the complex realities shaped by political and social contexts, public health strategies risk being overly generalized and less effective. Tailoring interventions to reflect the specific needs, assets, and contexts of diverse Latino subgroups enhances both the cultural relevance and the efficacy of cancer prevention and care initiatives.

A limitation of this report is the absence of direct, primary qualitative data from Latino patients or community members themselves, such as in-depth interviews or personal narratives. While the Task Force members bring lived experience and community-grounded understanding, future research should incorporate direct qualitative data collection to more fully capture the nuanced experiences, perspectives, and priorities of the diverse Latino population regarding cancer care and disparities.

TFLRAC seeks to create a more equitable healthcare landscape for Latinos, understanding that this is a progressive journey requiring concerted efforts from various sectors. Changing the course of cancer care among Latinos is a task that requires partnership, not only in academia, healthcare, and Latino communities, but in government and industry as well (56, 57).

## Data Availability

The original contributions presented in the study are included in the article/supplementary material, further inquiries can be directed to the corresponding author.
