# Correction: Uncovering multilevel drivers of cancer disparities among Latinos in the United States

**DOI:** 10.3389/fpubh.2025.1686736

**Published:** 2025-10-29

**Authors:** Amelie G. Ramirez, Edgar Munoz, Lorna Rodriguez-Rodriguez, Leon Bernal-Mizrachi, Patricia Chalela, Jose Aron Lopez, Paulo S. Pinheiro, Barbara Segarra-Vasquez, Gregory Talavera, Luis G. Carvajal-Carmona, Adolfo Diaz Duque, Cliff Despres, Edward J. Trapido

**Affiliations:** ^1^The University of Texas Health Science Center at San Antonio, San Antonio, TX, United States; ^2^City of Hope National Medical Center, Duarte, CA, United States; ^3^Emory University, Atlanta, GA, United States; ^4^University of Washington, Seattle, WA, United States; ^5^University of Miami, Coral Gables, FL, United States; ^6^University of Puerto Rico, San Juan, Puerto Rico; ^7^San Diego State University, San Diego, CA, United States; ^8^University of California, Davis, Davis, CA, United States; ^9^Louisiana State University School of Public Health, New Orleans, LA, United States

**Keywords:** cancer disparities, Latinos, health equity, cultural barriers, clinical trial diversity, environmental health

Author Patricia Chalela was omitted as an author in the published article. The correct author list reads:

Amelie G. Ramirez1^*^, Edgar Munoz^1^, Lorna Rodriguez-Rodriguez^2^, Leon Bernal-Mizrachi^3^, Patricia Chalela^1^, Jose Aron Lopez^4^, Paulo S. Pinheiro^5^, Barbara Segarra-Vasquez^6^, Gregory Talavera^7^, Luis G. Carvajal-Carmona^8^, Adolfo Diaz Duque^1^, Cliff Despres^1^ and Edward J. Trapido^9^

^1^The University of Texas Health Science Center at San Antonio, San Antonio, TX, United States

^2^City of Hope National Medical Center, Duarte, CA, United States

^3^Emory University, Atlanta, GA, United States

^4^University of Washington, Seattle, WA, United States

^5^University of Miami, Coral Gables, FL, United States

^6^University of Puerto Rico, San Juan, Puerto Rico

^7^San Diego State University, San Diego, CA, United States

^8^University of California, Davis, Davis, CA, United States

^9^Louisiana State University School of Public Health, New Orleans, LA, United States

The Author Contributions Statement has been corrected to read:

AR: Conceptualization, Data curation, Formal analysis, Funding acquisition, Investigation, Methodology, Project administration, Resources, Supervision, Writing – original draft, Writing – review & editing. EM: Conceptualization, Data curation, Formal analysis, Methodology, Project administration, Resources, Software, Visualization, Writing – original draft, Writing – review & editing. PC: Conceptualization, Data curation, Formal analysis, Methodology, Project administration, Writing – original draft, Writing – review & editing. LR-R: Writing – original draft, Writing – review & editing. LB-M: Writing – original draft, Writing – review & editing. JL: Writing – original draft, Writing – review & editing. PP: Writing – original draft, Writing – review & editing. BS-V: Writing – original draft, Writing – review & editing. GT: Writing – original draft, Writing – review & editing. LC-C: Writing – original draft, Writing – review & editing. AD: Writing – original draft, Writing – review & editing. CD: Visualization, Writing – original draft, Writing – review & editing. ET: Writing – original draft, Writing – review & editing.

The original version of this article has been updated.

